# Sensor-Based Optimized Control of the Full Load Instability in Large Hydraulic Turbines

**DOI:** 10.3390/s18041038

**Published:** 2018-03-30

**Authors:** Alexandre Presas, David Valentin, Mònica Egusquiza, Carme Valero, Eduard Egusquiza

**Affiliations:** Center for Industrial Diagnostics and Fluid Dynamics (CDIF), Polytechnic University of Catalonia (UPC), Av. Diagonal, 647, ETSEIB, 08028 Barcelona, Spain; alexandre.presas@upc.edu (A.P.); monica.egusquiza@upc.edu (M.E.); m.del.carmen.valero@upc.edu (C.V.); eduard.egusquiza@upc.edu (E.E.)

**Keywords:** hydraulic turbine, dynamic behavior, physical sensors

## Abstract

Hydropower plants are of paramount importance for the integration of intermittent renewable energy sources in the power grid. In order to match the energy generated and consumed, Large hydraulic turbines have to work under off-design conditions, which may lead to dangerous unstable operating points involving the hydraulic, mechanical and electrical system. Under these conditions, the stability of the grid and the safety of the power plant itself can be compromised. For many Francis Turbines one of these critical points, that usually limits the maximum output power, is the full load instability. Therefore, these machines usually work far away from this unstable point, reducing the effective operating range of the unit. In order to extend the operating range of the machine, working closer to this point with a reasonable safety margin, it is of paramount importance to monitor and to control relevant parameters of the unit, which have to be obtained with an accurate sensor acquisition strategy. Within the framework of a large EU project, field tests in a large Francis Turbine located in Canada (rated power of 444 MW) have been performed. Many different sensors were used to monitor several working parameters of the unit for all its operating range. Particularly for these tests, more than 80 signals, including ten type of different sensors and several operating signals that define the operating point of the unit, were simultaneously acquired. The present study, focuses on the optimization of the acquisition strategy, which includes type, number, location, acquisition frequency of the sensors and corresponding signal analysis to detect the full load instability and to prevent the unit from reaching this point. A systematic approach to determine this strategy has been followed. It has been found that some indicators obtained with different types of sensors are linearly correlated with the oscillating power. The optimized strategy has been determined based on the correlation characteristics (linearity, sensitivity and reactivity), the simplicity of the installation and the acquisition frequency necessary. Finally, an economic and easy implementable protection system based on the resulting optimized acquisition strategy is proposed. This system, which can be used in a generic Francis turbine with a similar full load instability, permits one to extend the operating range of the unit by working close to the instability with a reasonable safety margin.

## 1. Introduction

Hydropower has become a key renewable energy source in the last years due to its extreme regulation capacity which introduces flexibility in the electricity generation. This flexibility permits the use of other renewable sources, that are extremely unpredictable and volatile, such as wind or solar power. In this context, hydraulic turbines can be adjusted so that the total energy generation matches the energy demand at every instant. In order to achieve this purpose, hydraulic turbines have to work with reasonable safety and without excessive vibrations in a very extensive operating range [1]. 

Francis turbines, are by far the most used turbine type due to their wide range of design heads (from a few tens to several hundreds of meters [2]). Nevertheless, compared to other types of units, such as Kaplan or Pelton, Francis units working under off-design conditions suffer from many types of problems and damages. Besides, the erosive cavitation [3,4,5] and fatigue problems [6,7,8,9,10], which have been extensively discussed, one of the critical problems are the power swings [11,12]. The work of Rheingans [11], written many years ago, reports a power swing in a real turbine and may be one of the first studies regarding this problem. Power swings can occur under off-design conditions, below or above the best efficiency point (BEP), when the vortex rope emerges. The vortex rope appears at the draft tube (outlet of the turbine) and acts as an exciter of the entire hydraulic circuit. If the excited frequency, that depends on various operating factors, coincides with a hydraulic natural frequency of the hydraulic system a resonance may occur, producing high power swings [12,13,14,15,16,17,18]. 

**Figure 1:**
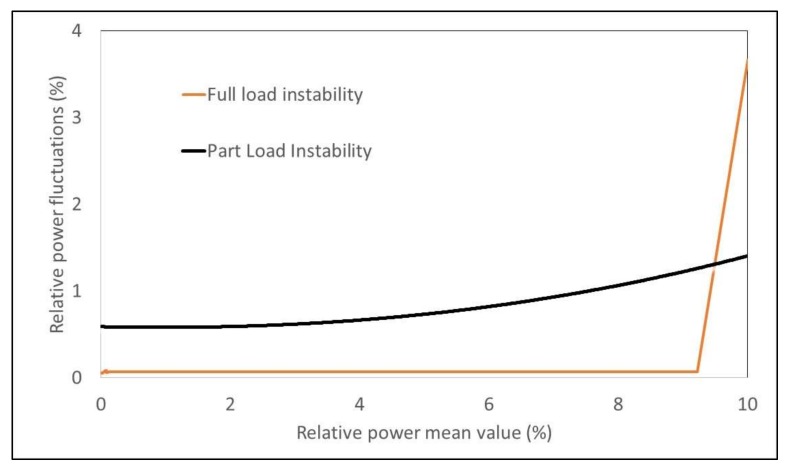
Relative power fluctuations before reaching the part load instability and the full load instability.

There are mainly two well differentiated types of vortex ropes in the whole operating range of a Francis unit. The first one, the part load vortex rope [13,14,15,16], occurs below the best efficiency point and it is defined by a clear precessing motion of the core. The full load occurs above the best efficiency point (the flow-rate is larger than for the BEP) and it is characterized by a more centered and stable core. In the past, several instabilities have been observed for both, the part load vortex rope [13] and for the full vortex rope [12,17,18]. Nevertheless, the onset of the full load instability is usually much more abrupt and less progressive than the part load vortex rope. This means, that the machine can operate very close to this instability with low vibrations and low pressure fluctuation levels (Figure 1). 

The behavior of the full load instability is much more dangerous than the behavior from the part load instability, because a small change in the operating condition can lead the machine to an unstable condition. For this reason, some power plants prefer to avoid this situation by operating far away from this condition [19,20,21]. Nevertheless, this reduction of the operating range implies a huge reduction on the capacity of the machine to produce energy, a less efficient use of renewable energies and economic losses for the operators.

This situation could be improved by using a sensors-based control system, that rapidly detect the onset of such instability before it is fully developed and consequently acts on the unit governing system. Within the framework of a large EU project (Hyperbole [22]) one of the biggest Francis units in Canada was investigated. The unit has a rated power of 444 MW and has served for many research purposes [12,23]. Related to the topic of the full load instability, the work performed by Müller et al. [17,18,24] in the reduced scale model have helped to improve the understanding of the physical phenomena that involves this instability. In the aforementioned studies performed in a reduced scale model, the full load instability has been analyzed and observed by means of high speed camera and advanced visual techniques such as Laser Doppler Vibrometer (LDV), or Particles Image Velocimetry (PIV) combined with pressure sensors measurements and torque measurements. These visual techniques, which unfortunately cannot be used in the prototype, have permitted an improved understanding of the physical phenomena occurring in the model. More recently, Presas et al. [25] briefly compared the phenomena observed on the reduced scale model and the prototype. Valentin et al. [12] have also shown, by comparing experimentation and a numerical simulation model, that the planar waves generated by the vortex rope during the instability, produce a torsion on the runner and consequently torque and electrical power fluctuations. Finally, Presas et al. [26] showed a general description of the sensors installed during the tests that were capable to detect this instability. 

Based on the preliminary conclusions of that study, this paper is focused on an advanced analysis of the sensors’ behavior during the onset of this instability. The main objective is to discuss and analyze which sensors are more reactive and more feasible to anticipate the onset of the full load instability. This include not only the type and number of sensors, but also the acquisition frequency of the sensors and corresponding signal processing to obtain reliable indicators. In this way, the best indicators to anticipate the onset of the instability are proposed. Finally, based on this optimized acquisition strategy, a scheme for the design of a sensors-based control system to protect a generic Francis unit of reaching this type of instability is proposed. In this way, the operating limits of the unit can be more accurately defined and therefore the effective operating range can be increased, which results in a safer operation of the machine, more effective use of renewable energies and more economical benefits for the machine operators.

## 2. Full Load Instability in the Francis Turbine and Experimental Set-Up

### 2.1. General Description of the Unit and the Phenomena

**Figure 2:**
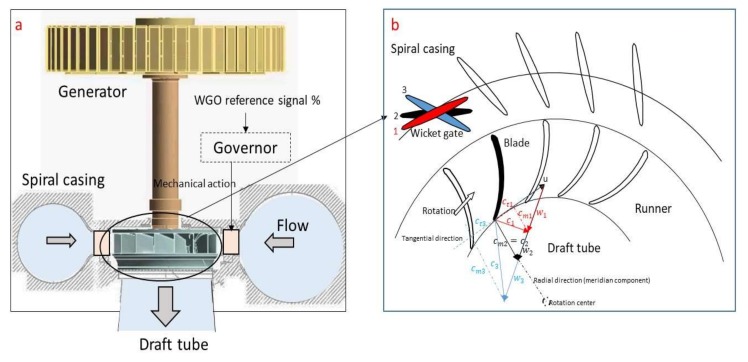
General description of the analyzed unit (**a**) and detail view of the runner velocities at the outlet of a generic Francis runner (**b**).

In Figure 2a a general view of the Francis unit studied and its main components are shown. The unit has a rated power of 444 MW. The turbine has 16 blades, whereas the distributor has 20 guide vanes. The rotating speed of the machine is 128.6 rpm (2.14 Hz). As in every Francis turbine, the water flows from the spiral casing through the runner into the draft tube. When the water passes through the runner, most of its kinetic and pressure energy is transformed into mechanical energy that is used to generate electricity in the generator. To adjust the electrical power generated, the flow rate Q has to be varied. This is done by adjusting the Wicket Gate Opening (WGO). Briefly described, the wicket gates are moved by the mechanical action of the governor, which transforms an electrical input (WGO reference signal) into the corresponding mechanical action that adjusts the position of the wicket gates (Figure 2a). 

Figure 2b shows the adjustment of the position of the wicket gates in a generic Francis runner. For clarity in the representation, only one wicket gate interacting with only one blade of the runner is shown. The velocity triangle at the runner outlet (entrance on the draft tube) is represented for three different wicket gate openings. *u* corresponds to the speed of the rotating system, *w* corresponds to the relative velocity to the rotating system and *c* to the absolute velocity, so that $\vec{c}=\vec{u}+\vec{w}$. 

To analyze the appearance of the vortex rope it is important to analyze the tangential component of the velocity at the outlet. Position (2) corresponds to the design condition or Best Efficiency Point, where the absolute velocity exits the runner with no tangential component. Position (1) represents a partial load condition, where the flow rate Q is smaller and therefore the meridian velocity has to decrease. As a consequence, *c* has now a tangential component *c_t_* in the same direction than the rotation. Finally, in position (3) or full load condition, the meridian velocity increases and *c* has a tangential component *c_t_* in the opposite direction than the rotation.

As a consequence of these tangential components at part load and full load, a vortex rope in the draft tube can appear. Physical conditions for the appearance of this rope can be found in many references such as [17,18,27,28]. In the full load case (3), the meridian component is much greater than the tangential component (Figure 2) and therefore a centered vortex rope with almost no precession of the core (motion of the core around the rotation center) appears. In the part load case (1), both components have a similar magnitude order and therefore the rope is characterized by a greater precession.

Both ropes are capable to generate synchronous pressure pulsations that are propagated in all the hydraulic circuit. The frequency of these pulsations, which are around (0.2–0.5 *f_rot_*), changes slightly with the operating conditions of the machine, such as wicket gate opening and outlet pressure. For certain conditions, a coincidence between the pulsations generated by the vortex rope and an acoustic frequency of the hydraulic circuit may occur which will greatly amplify them, making all the systems (hydraulic, mechanical and electric) unstable. 

**Figure 3:**
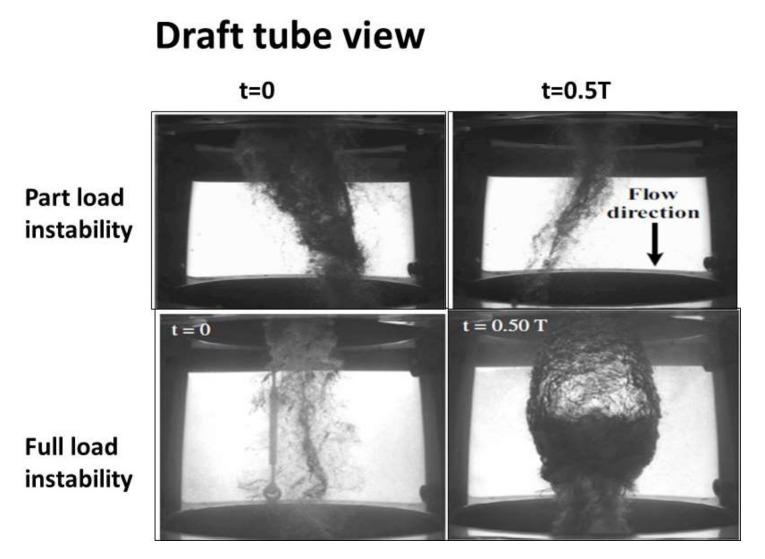
Vortex rope characteristic at the Turbine outlet during the part load and the full load instability

Figure 3 shows the draft tube vortex rope characteristic for the part load unstable condition and the full load unstable condition. It is shown at t = 0 and t = 0.5 T, where T is the characteristic period of the oscillation. At part load, the precession is clearly observed. The fact that a synchronous pressure pulsation (flow direction) exists can be clearly appreciated in both cases, as the volume of cavitation extremely varies, when comparing the picture at t = 0 and t = 0.5 T. These pictures have been obtained in the reduced scale model of the unit in the extensive work made by Müller et al. [17,18,24] and Favrel et al. [13,14,15,16].

### 2.2. Sensors Installed and Acquisition Strategy

Details of all the sensors used for these tests and different operating conditions tested can be found in [12,25,26]. Particularly in [26], a detailed discussion of the several types of installed sensors and the feasibility to use them for detecting the full load instability was performed. In this paper, we only discuss and show the most representative and sensitive sensors for this purpose, based on the preliminary conclusions of that analysis. 

**Figure 4:**
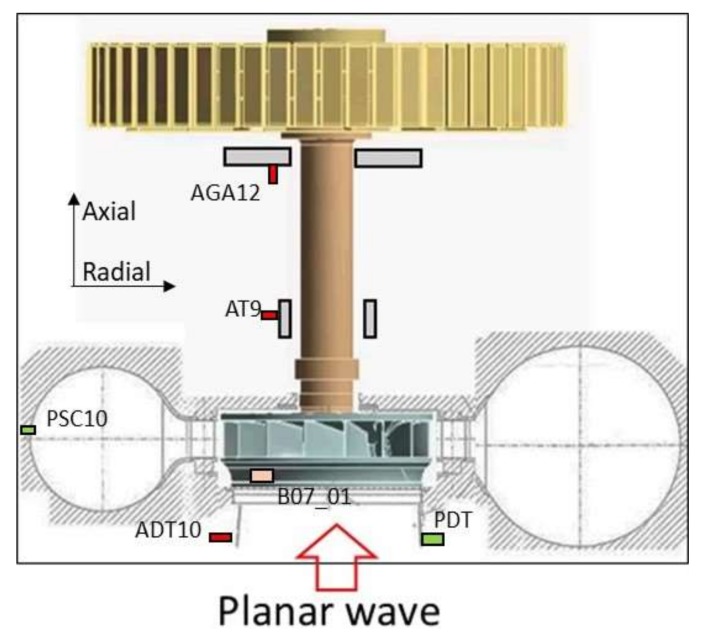
Sensors used for the full load instability analysis.

The sensors used for the present analysis are shown in Figure 4. The main characteristics regarding the type and location can be found in Table 1. All these signals were simultaneously acquired with a LAN XI Type 3053 module (Bruel & Kjaer, Naerum, Denmark) and with an acquisition frequency of 4096 samples/s. In order to analyze the cavitation phenomenon (high frequency content [3]) the accelerometer on the ADT10 draft tube was acquired in an independent channel with an acquisition frequency of 51,200 samples/s. 

In the present study, the power signal was also available. Usually, these signals can be picked up from existing control systems in the power station avoiding to install power meters in the generator. The main drawback of using such control signal of the power station is that usually the real analogical signal is resampled to a few samples per second. In this case, the used signal had an approximately sampling frequency of 5 samples/s. Fortunately, as the machine turns in a relative low rotation speed and the fluctuations are expected at 0.2–0.5 *f_rot_* (vortex rope), these were not filtered. Nevertheless, for machines with a faster rotating speed, these fluctuations may be filtered if the same type of signal is used, making it useless. This adds a justification and a utility of the present study especially for high rotating speed units where fluctuations caused by the vortex rope may be filtered in the power signal. 

## 3. Signal Analysis

The signal analysis techniques used in this paper for the analysis of the full load instability are briefly described in this section.

### 3.1. RMS Values

Root Mean Square (RMS) value is an indicator of the oscillating characteristic of any kind of time signal, as it considers the deviation with respect a mean value. Therefore, when the DC component of the signal is filtered (mean value 0) the RMS describes the intensity of the dynamic part of the signal. When the full load instability appears, the mean values of many signals do not substantially change but the RMS will increase due to the oscillating nature of the phenomenon (Figure 1). Mathematically, the RMS is described as:

(1) $$x_{RMS}=\sqrt{\frac{1}{n}\left(x_{1}^{2}+x_{2}^{2}+\ldots +x_{n}^{2}\right)}$$

where *n* is the number of samples in the part of the signal considered and $x_{i}$ the corresponding samples of the signal.

### 3.2. FFT Analysis

The Fast Fourier Transform is the classical transform to determine the frequency content of a time signal. Generally described, the FFT transforms a discrete time signal of *n* samples of the time domain into a signal of *n* samples in the frequency domain:

(2) $$X\left(f\right)=\mathrm{FFT}\left(x\left(t\right)\right),\;\mathrm{so}\;\mathrm{that}\;f_{i+1}-f_{i}=\frac{1}{t_{n}-t_{1}}$$

A more detailed description of the FFT can be found in many references, for instance [29]. The value of each X(f) represents the oscillating characteristic of the signal at that frequency. The vortex rope frequency in a particular machine, can be easily determined with simple tests [3]. In most of the cases, this value is empirically found in the range 0.2–0.5 *f_rot_*, where *f_rot_* is the rotating speed of the unit in Hz. 

The value of the characteristic frequency of the vortex rope *f_rope_*, slightly varies depending the operating conditions of the machine as shown by Favrel et al. [13]. If a full load instability occurs, its oscillating frequency will be the vortex rope frequency, so that $f_{rope}=f_{acoustic}$, where $f_{acoustic}$ is one of the natural frequencies of the hydraulic circuit. Therefore, to follow the variation of the oscillating characteristics of the signals, related to a possible onset of the full load instability it is proposed to follow the maximum value in a frequency band of the signal X(f) that includes the vortex rope in all the situations, i.e., the interval $\left[f_{min}:f_{max}\right]$ has to contain $f_{rope}$ in all the operating conditions of interest. The indicator, for every analyzed signal, is obtained according to Equation (3):

(3) $$x_{FFT-rope}=\max\left[X\left(f_{min}\right):X\left(f_{max}\right)\right],\;\mathrm{with}\;f_{rope}\in [f_{min}:f_{max}]$$

### 3.3. Envelope Characteristic Frequencies with Hilbert Transform

The Hilbert transform of a time signal can be used to determine its envelope. The idea is that, if the full load instability exists, the time signals will appear modulated with the characteristic frequency $f_{rope}$. This idea is similar as the case of the detection of erosive cavitation, that has proven to be effective in the past [3].

Mathematically, the Hilbert transform of a time signal can be described as:

(4) $$Hi\left(x\left(t\right)\right)=\frac{1}{\pi }\int_{-\infty }^{\infty }x\left(\tau \right)\frac{1}{1-\tau }d\tau$$

and from this signal, the envelope amplitude can be obtained as:

(5) $$\tilde{x}\left(t\right)=\sqrt{x\left(t\right)+Hi{\left(x\left(t\right)\right)}^{2}}$$

$\tilde{x}\left(t\right)$ describes then the envelope of the signal x(t). Therefore, the FFT technique and procedure explained in the previous section can be used in the same way but now applied to $\tilde{x}\left(t\right)$ to obtain the indicator $x_{envelope-rope}$, for every analyzed signal.

### 3.4. Time-Frequency Analysis with Wavelet

The Time-Frequency using Wavelets is a powerful analysis tool to study short transient phenomena and determine its instantaneous frequency content. In such cases, where the signal is not periodical, the information obtained with a time frequency analysis can be much useful and conclusive than the information obtained with the FFT analysis [30]. Details of this transform can be found in many references [31,32,33,34]. 

Wavelets have the general expression:

(6) $$\psi_{s,\tau }\left(t\right)=\frac{1}{\sqrt{s}}\psi \left(\frac{t-\tau }{s}\right)$$

where *s* scales the frequency of the wavelet and τ translates the wavelet in time. ψ is the mother wavelet used. In this case the Morlet wavelet is chosen (see [31] for more details on this wavelet). Results of this transform will be presented in a 2-dimensional contour plot. 

In the context of the present study, the information obtained with this analysis will be used qualitatively to confirm the existence of the full load instability in a similar way that exists in the model [24] as the vortex rope cannot be physically observed in the prototype. Nevertheless, the computational capacity necessary and complexity of this analysis, makes it less suitable for a continuous monitoring of the instability onset.

## 4. Results and Analysis of an Optimized Acquisition Strategy

The main purpose of this section is to see which type of sensor combined with suitable signal processing is more sensitive to detect and advance the onset of the full load instability before it is fully developed. This is a critical point and the most relevant objective of the present research, since the unit can work very close to the onset of the full load instability, without showing excessive vibrations, pressure fluctuations or power fluctuations. In fact, in the particular case analyzed, the machine is working in a very smooth condition before the onset of the instability (Figure 1). Therefore, in order to work close to the instability with a reasonable safety, extending the operating range of the unit, an accurate signal analysis of the different sensors during the onset of the instability is necessary. 

### 4.1. Analysis of the Instability: Comparison with the Reduced Scale Model

**Figure 5:**
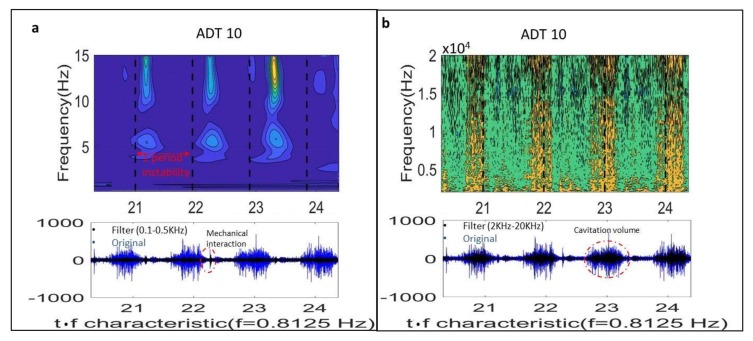
Sensor ADT-high frequency. Mechanical phenomenon of the pressure wave (**a**) and volume of cavitation oscillating (**b**).

Firstly, it is checked that the instability observed and measured in the prototype corresponds to the instability found in the model. The full load instability is characterized by two related but well differentiated physical phenomena, according to the experiments made in the model (Figure 3). The first phenomenon is characterized by a huge volume of cavitation oscillating. For t = 0.5 T *(*Figure 3) the cavitation volume is at its maximum and generates high frequency noise and vibration that can be clearly detected by the sensor ADT acquiring at high frequency. Figure 5b shows the high frequency components of the signal (2 kHz–20 kHz). In the Time-Frequency plane, bands involving the high frequency components of the signal can be appreciated every cycle of the instability. In the time signal, this is seen by the black signal (high pass filtered signal) overlaying the blue signal (original signal). This zone has a progressive increasing until a maximum (volume of cavitation at its maximum and closer to the sensor) and further progressive decreasing until a minimum (t = 0 T in Figure 3). This filtered signal perfectly describes the behavior of the cavitation volume observed in the model (Figure 3). 

**Figure 6:**
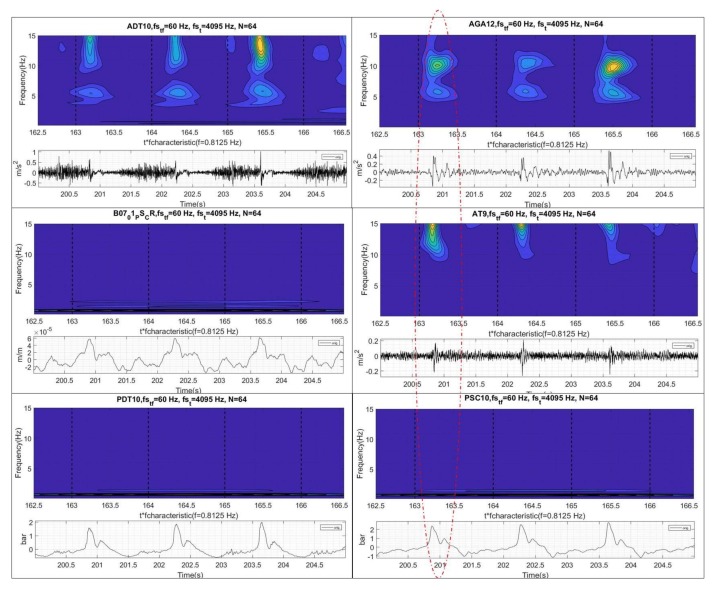
Time and time-frequency analysis of the different sensors.

The second phenomenon is the planar wave that travels axially from the draft tube and propagates to the spiral case at the speed of sound. Before the wave reaches the spiral case, it passes through the turbine runner twisting it as discussed in [12]. The rest of the sensors clearly detect the interaction of the planar wave with the mechanical system (Figure 6). As the propagation speed is very high, the peak observed in the pressure sensors occurs approximately simultaneously with the peak observed in the sensors on the mechanical system (see peaks in all sensors of Figure 6). This “impact” excites the low frequencies of the mechanical system (once every cycle) as seen in the time-frequency plots of the same figure. Note that these sensors are acquiring at a low frequency and do not detect the cavitation behavior described with the sensor ADT acquiring at high frequency. 

**Figure 7:**
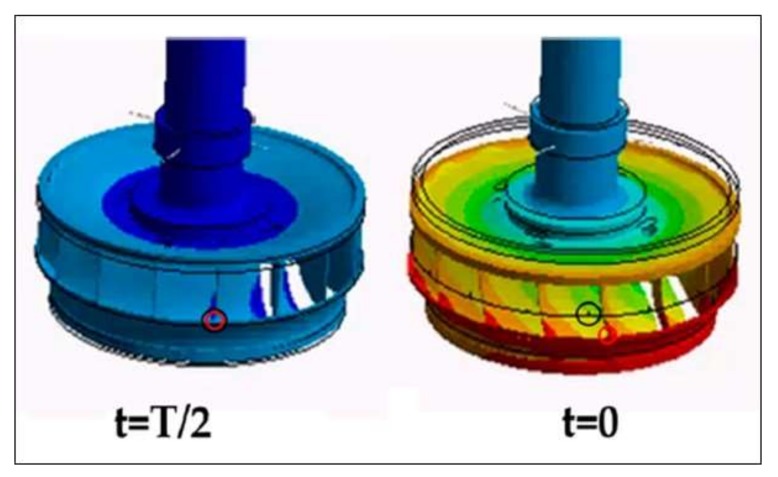
Torsion of the runner when the planar wave (peak of pressure) passes through it (t = 0).

Figure 5 helps to see the interaction between the cavitation volume and the planar wave. As the planar wave passes through the draft tube, it increases the pressure reducing the volume of cavitation. Approximately at the same time when the volume is at its minimum and the pressure is at its maximum, the interaction of the planar wave with the mechanical system occurs, i.e., the “impact” on the mechanical system occurs approximately at (t = 0 T in Figure 3 and Figure 7) when the cavitation volume is at its minimum and the twisting of the runner is approximately at its maximum (see mark on Figure 7).

### 4.2. Time and Time-Frequency Characteristic of the Instability Onset

**Figure 8:**
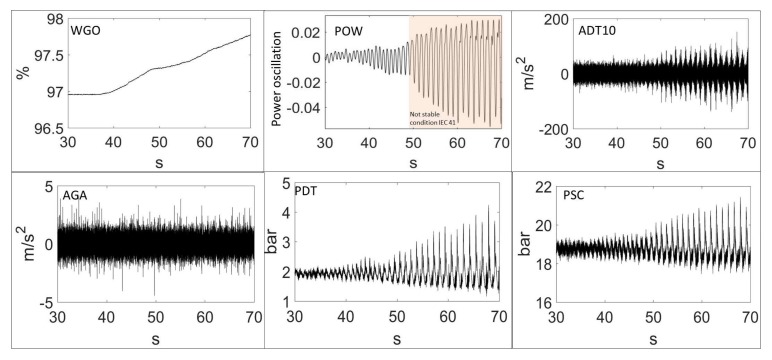
Time signals during the onset of the instability.

The transition of the different signals from a stable situation (inside the limits defined by the standard IEC60041) to an unstable behavior is shown in Figure 8, for the different sensors used. In the power signal, the scale is obtained by making the amplitude relative to the mean value before removing the continuous component. Therefore, the relative oscillating power is shown. As seen in this figure, the oscillating power at the beginning is less than 1%, which is inside the admissible range defined by the IEC41. The wicket gate opening (WGO) remains constant during approximately the first 7 s. After that, in order to force the instability, the WGO is slowly increased. Then, the oscillating power increases to almost 7% (which corresponds to an oscillation of approximately 30 MW). Under these conditions, the machine is fully unstable and a continuous operation is not acceptable for the electrical grid stability and for the safety of the power plant.

**Figure 9:**
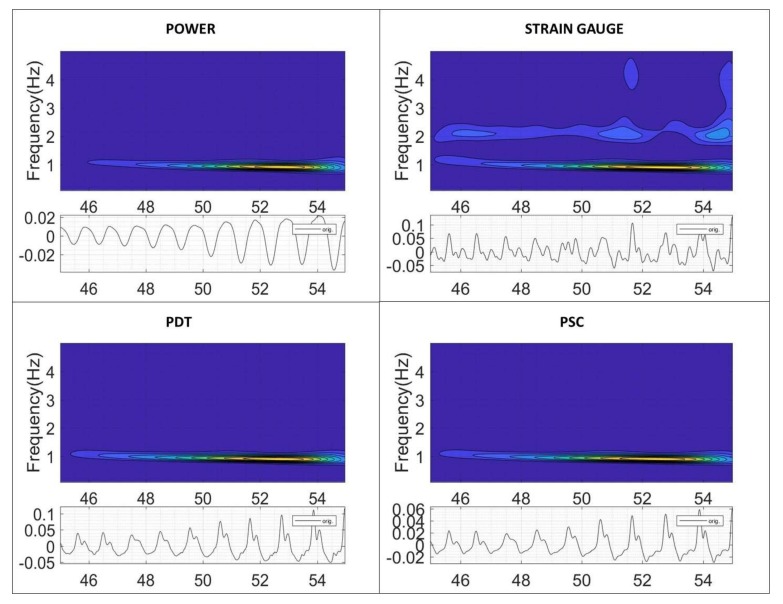
Onset of the instability. Time (s) and time-frequency analysis.

The deeper analysis performed in Figure 9 shows how the instability appears. As seen in this figure, the excited pressure pulsations by the vortex rope slightly reduces their frequency (from about 1 Hz to 0.85 Hz) as the WGO increases (Figure 8 for the range 45–55 s). 

At the same time, the oscillating amplitude increases as the frequency gets closer to an acoustic frequency of the hydraulic part and the whole unit behavior becomes unstable. In this figure, the hydraulic part is characterized by the pressure sensors, the mechanical part by the strain gauge on the runner and the electrical part by the generated power. Now, it is desired to see which type of sensor and signal analysis can better describe the onset of this instability. 

### 4.3. Sensitivity to the Detect the Instability Onset

An optimized acquisition strategy to detect the onset of the instability with high sensitivity and reactivity will be discussed next. The resulting strategy will be used in the following section to design a protection system that prevents the unit from reaching a fully developed instability. Acquisition strategy means not only the selection of the appropriate sensor, but also an appropriate signal acquisition frequency, windowing and corresponding analysis. 

In the present test, the power signal is available for the tests and the oscillating frequency of the instability is small enough to not be filtered by the resampling of the operating signals used in the power station. Nevertheless, as mentioned before, the objective is to determine an acquisition procedure, which can supply this information in a reliable way and independently from the operating signals of the unit. Consequently, it is desired to find a procedure that can be used in a generic case. 

**Figure 10:**
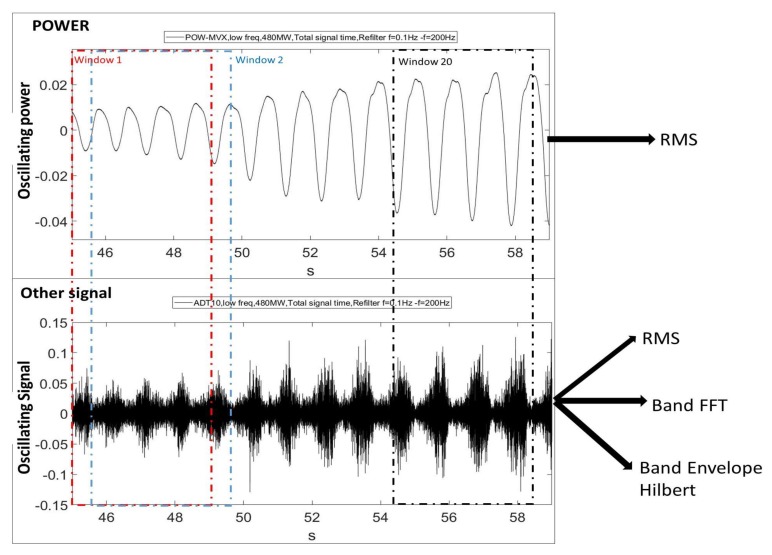
Evaluation of the acquisition strategy.

To quantitatively evaluate and determine which the best acquisition strategy is, the oscillating power, shown in Figure 10 is considered. At the beginning of the signal, small power oscillations are present (stable condition according to IEC 41). Nevertheless, the oscillations continuously increases during this time, as discussed in Figure 8 and Figure 9, until the unit becomes fully unstable. The signal is windowed with 4 s Hanning windows. No shorter windows are recommended as a precision in frequency of at least ¼ Hz is necessary for the analysis of $f_{rope}$.

For every window (sample), the RMS value of the oscillating power is calculated ($x_{RMS-power})$. At the same time, for the rest of the sensors, the RMS value ($x_{RMS})$, the amplitude of $f_{rope}$ based on FFT analysis ($x_{FFT-rope})$, and the amplitude of $f_{rope}$ based on the Hilbert analysis ($x_{Envelope-rope})$ is calculated (see Section 4). After these indicators are calculated for one part of the signal, the window is shifted 0.5 s and all these indicators are newly calculated. In total twenty samples or windows are used for the analysis of the instability onset. The time of every sample corresponds to the time when the window starts. All the signals are normalized to its maximum amplitude (the power is normalized with the mean value) and then, the mean value is filtered in order to have comparable correlations in all the cases independently from the sensor type. The correlation between the indicators obtained for all the sensors and the RMS value of the oscillating power ($x_{RMS-power})$, will be shown in the following paragraphs.

#### 4.3.1. RMS Indicators

**Figure 11:**
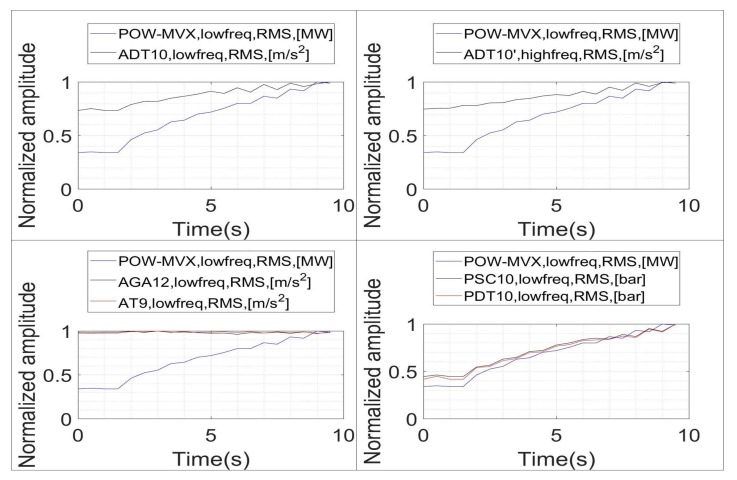
Evolution of the RMS indicators during the onset of the instability ($x_{RMS-power}$ vs. $x_{RMS}$).

The evolution of the RMS values of all the signals during the onset of the instability, including the power, is shown in the plots of Figure 11. A good indicator should increase at the same time that $x_{RMS-power}$ increases. This is generally observed for all the sensors except for AGA-12 and AT9, whose RMS values remain approximately constant.

**Figure 12:**
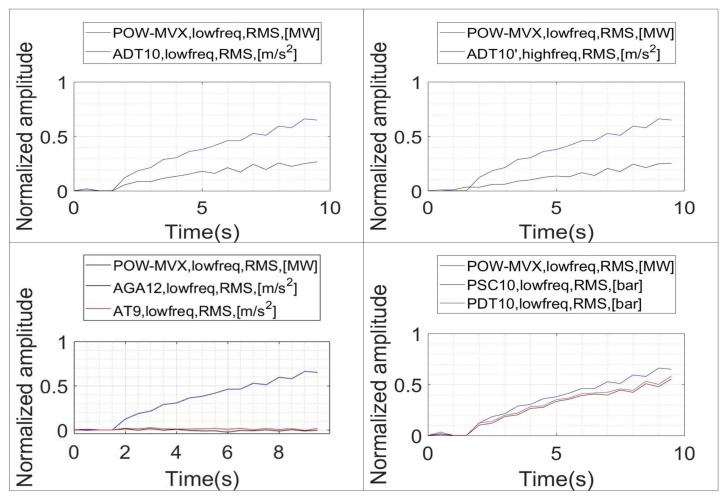
Evolution of the RMS indicators during the onset of the instability. Shifted trend according to initial value.

**Figure 13:**
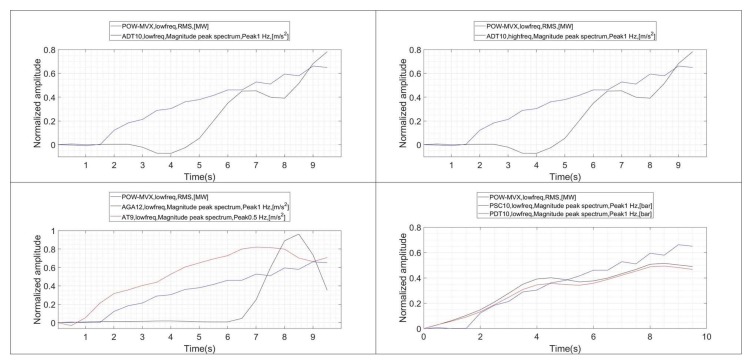
Evolution of the band $f_{rope}$ on the FFT and RMS of the power signal ($x_{FFT-rope}$ vs. $x_{RMS-power}$).

**Figure 14:**
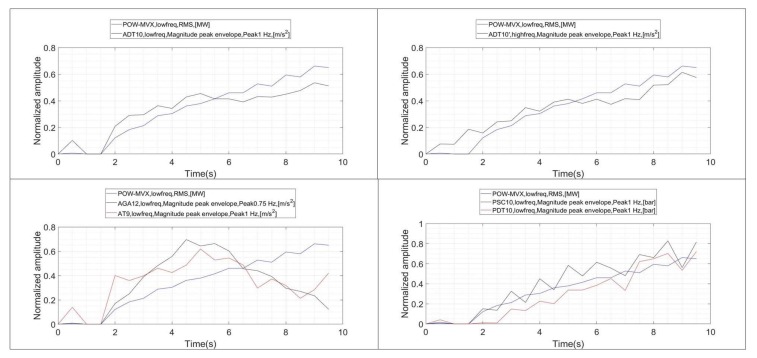
Evolution of the band $f_{rope}$ on the Envelope and RMS of the power signal ($x_{Envelope-rope}$ vs. $x_{RMS}$).

In order to improve this representation, all the graphs are shifted so that the initial value corresponds to 0 (Figure 12). This will be done also for the following graphs (Figure 13 and Figure 14). This shift allow us to show the correlation between the different indicators and $x_{RMS-power}$ in a better way. In the case of the RMS values obtained for the different sensors (Figure 12), it is appreciated that the two pressure sensors exhibit an excellent correlation with increasing $x_{RMS-power}$. They have also a higher relative variation rate than the rest of the sensors. No differences can be appreciated between calculating the RMS value in ADT with high frequency or low frequency acquisition. Finally, the RMS values of the sensors AGA12 and AT9 do not show an increasing behavior when $x_{RMS-power}$ starts to increase, indicating a poor correlation. 

#### 4.3.2. FFT Band

The same type of analysis is performed to correlate $x_{RMS-power}$ with the indicators obtained with the vortex rope band on the FFT ($x_{FFT-rope}$). This correlation is shown in Figure 13. Now, only the pressure sensors show a good correlation with respect to t$x_{RMS-power}$. Furthermore, with respect the previous indicators, in this case $x_{FFT-rope}$ of the pressure sensor start to increase before $x_{RMS-power}$ does. This is an advantage, since it shows that they can detect the onset of the instability before the power starts to oscillate. 

#### 4.3.3. Envelope through Hilbert Transform

The evolution of $x_{Envelope-rope}$ and $x_{RMS-power}$ is shown in Figure 14. Now, a positive correlation characteristic is observed for the pressure sensors and ADT. The highest linear correlation might be seen for the sensor ADT acquiring at high frequency, which is normal considering that the envelope characteristic is enhanced when a larger frequency band is considered. 

#### 4.3.4. Linear Regression and Optimization

**Figure 15:**
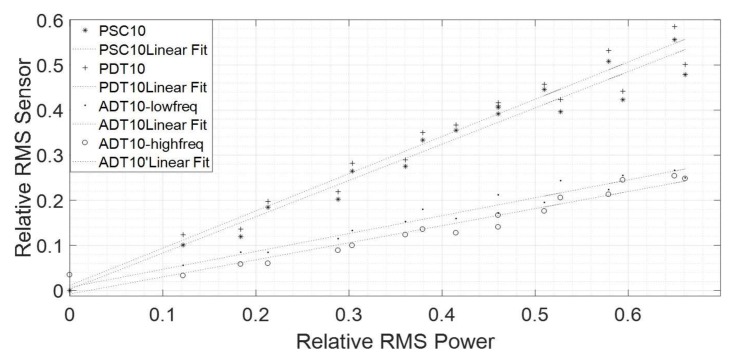
Correlation $x_{RMS-POWER}$ and $x_{RMS\;}$ for the different signals.

Based on the previous indicator trends, now the quality of the correlation may be evaluated considering the following characteristics or desirable features of the correlation (in order of importance):

- Linearity with respect to $x_{\mathrm{RMS}-\mathrm{power}}$: It is desirable that the indicators follow a linear correlation with the $x_{RMS-power}$ in order to simplify the control model of the instability and in order to predict the oscillating power (Figure 15).
- Slope of the correlation $x_{\mathrm{RMS}-\mathrm{power}}$ vs. Indicator: Higher slopes will indicate a more sensitive indicator to detect a variation in the RMS of the oscillating power.
- Advancement with respect increasing power: If the two preceding indicators are achieved a third indicator is the advancement, which helps to advance the detection of the instability (reactivity). Graphically it can be seen as the value of the indicator, when $x_{RMS-power}$ starts to increase (approximately at t = 1.5 s in Figure 12, Figure 13 and Figure 14).

The well-known statistical techniques for simple linear regression are used. The independent variable is considered as $x_{RMS-power}$ and the dependent variable the different values of the indicators ($x_{RMS},x_{FFT-Rope},x_{Envelope-Rope}$). In this phase of the analysis, AGA12 and AT9 have been excluded due to the poor correlation observed (Figure 12, Figure 13 and Figure 14).

The simple linear regression equation with $x_{RMS-power}$ as independent variable, can be described as:

(7) $${\tilde{x}}_{inidcator}=s\cdot x_{RMS-POWER}+a$$

where ${\tilde{x}}_{inidcator}$ is the estimated value of the indicator. s indicates the slope and is a measurement of the sensitivity with respect to a change $x_{RMS-POWER}$. a is the intercept and is a measurement of the advancement with respect $x_{RMS-POWER}$ or reactivity.

The classical $R^{2}$ parameter shows the quality of the linear regression, i.e., how much of ${\tilde{x}}_{inidcator}$ can be explained by the linear model. It is obvious, that the correlation can be easily reformulated exchanging the independent and dependent variables without affecting $R^{2}$.

As an example, Figure 15 shows the correlation between $x_{RMS-POWER}$ and the $x_{RMS}$ for the rest of the signals. All of them show a good linear correlation. The pressure sensors show a relatively high slope (more sensitive to detect a change in $x_{RMS-POWER\;}$). No clear advancement of the indicators is appreciated (intercept a > 0).

All the correlations between indicators and $x_{RMS-POWER\;}$ are summarized in a single table (Table 2). As seen in this table, for the four sensors selected, the RMS values ($x_{RMS\;})$ correlate in a linear matter with $x_{RMS-POWER\;}$ ($R^{2}$ close to 1), the slope is higher for the pressure sensors (also seen in Figure 15) and no significant intercept is observed. 

Regarding the FFT indicators $x_{FFT-rope}$, the pressure sensors exhibit also a high linear correlation and with respect to the previous indicators they show a positive intercept, which quantifies reactivity of these indicators. The accelerometer ADT does not show a good correlation as such a frequency band ($f_{rope}<1\;\mathrm{Hz})$ is too low for an IEPE accelerometer.

Finally, regarding the Envelope analysis, although all the sensors show a moderate good correlation factor ($R^{2}$ > 0.8), the sensor on the draft tube acquiring at high frequency shows a very high $R^{2}$ factor, a relatively high slope and a significant positive intercept. 

Based on these results, the optimized acquisition strategy is selected choosing between the following indicators, which all of them show excellent correlation features with respect $x_{RMS-power}$.

- $x_{RMS}$ of the pressure sensors,
- $x_{FFT-rope}$ for the pressure sensors,
- $x_{Envelope-rope}$ for ADT-highfreq.

The main drawback of ADT-highfreq. is the much higher acquisition frequency necessary (51.2 kHz in the present case) to obtain $x_{Envelope-rope}$. For the implementation of the protecting device, this will require a much more powerful computational capacity of the hardware, which will be more prone to suffer some failures. Finally, this indicator does not provide a significantly advantage regarding the other two options. Therefore, this option is discarded. 

Between $x_{RMS}$ and $x_{FFT-rope}$ of the pressure sensors, which have both an excellent linear correlation with $x_{RMS-Power}$, the use of $x_{FFT-rope}$ seems to be more advantageous. Although the relative slope is smaller, the intercept is significantly greatly than 0, which shows a capacity to advance the onset of the instability. Furthermore, the reason is also from the acquisition frequency; although in this case the acquisition frequency was the same to calculate both indicators (4096 Hz), for the implementation of a protecting device, the $f_{rope}$ band can be accurately characterized using an acquisition frequency of $f_{acquisition}>2.56\cdot f_{rope}$ [35]. For the present case this would mean an acquisition frequency of $f_{acquisition}\approx 3\;\mathrm{Hz}$, much lower than the acquisition frequency necessary to obtain $x_{RMS}$. 

**Figure 16:**
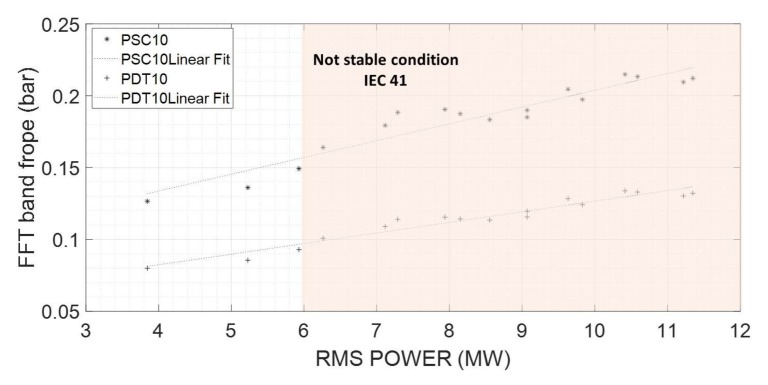
Optimized acquisition strategy. Correlation of indicators.

Due the physical nature of the phenomena, which is a planar wave propagating from the draft tube into the spiral case, it is very interesting to use both pressure sensors (spiral case and draft tube) instead of one. The reason is that if both sensors are showing a high $x_{FFT-rope}$ value, the most probable physical explanation is that a planar wave is propagating through the entire hydraulic circuit, causing a high torsion in the turbine and therefore a fluctuation in the electrical power. The linear correlation between the resulting indicators $x_{FFT-rope}$ of the pressure sensors and $x_{RMS-power}$ is shown in an absolute scale in Figure 16. 

**Figure 17:**
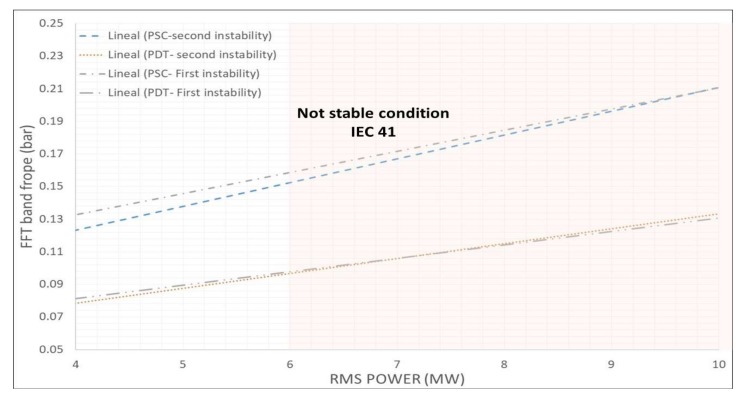
Optimized acquisition strategy. Repeatability of the indicators.

Finally, in order to prove the repeatability and robustness of the indicators selected, a second case of full load instability in the same unit is analyzed for comparison. This second case was tested on the same day, several minutes after the first case used in all this analysis. Again, departing from a stable condition, the wicket gate opening was slightly increased until reaching the instability. Applying the procedure explained in this section, the correlation between indicators is calculated and shown in Figure 17. As seen in this figure, in both tests, the correlation between the $x_{FFT-rope}$ of the pressure sensor and $x_{RMS-power}$ are almost identical, which reinforces the selection of this acquisition strategy.

## 5. Proposed Protection System to Increase the Operating Range of the Unit

Based on the acquisition strategy discussed in the previous section, a protection system to increase the operating range of the unit is proposed. This system permits one to work close to the full load instability with safety (Figure 1). The actual unit does not have such specific protecting systems (it works as shown in Figure 2) and in order to avoid the full load instability, the turbine works far from this condition, reducing its effective operating range and energy generated during the year [19]. The proposed system considered here can work in a generic unit with a similar instability phenomenon and independently from the electrical power signal due to the linearity shown in the previous section. 

**Figure 18:**
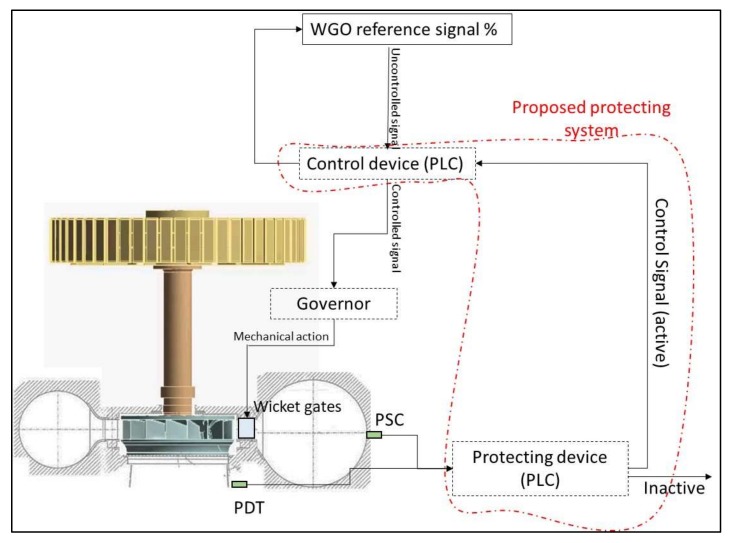
Scheme for the full load instability protecting system.

The system consists of two standard piezo resistive pressure sensors and two PLC devices (Figure 18). Ideally, the pressure sensors have to be flush mounted on the spiral case and draft tube surface to not filter any frequency. The first PLC (Protecting device) implements the calculation of $x_{FFT-rope}$ as explained in the previous section. If both values, corresponding to the two pressure sensors, exceed the respective threshold levels a control signal is activated that corrects the original WGO reference (reduces the WGO signal send to the governor) until a stable condition is reached and the control signal is inactive again. 

Note that the instability can appear during a load change to increase the power, therefore increasing WGO reference signal, but also for a stable WGO reference signal and a change of the operating head or cavitation number [13]. The system has to protect the unit from reaching this instability in both situations. 

**Figure 19:**
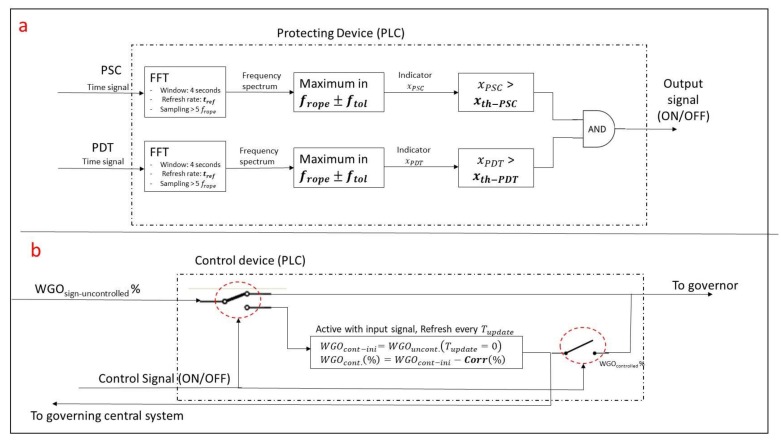
(**a**) Scheme of the protection device. (**b**) Scheme of the control device.

Figure 19a shows how the protection device (PLC) works. Basically it implements the calculation of the indicators according to the previous section. Windows of 4 s in order to have good frequency resolution are selected. Every 0.5 s the window is refreshed performing a new calculation. The sampling frequency has to be high enough to clearly define the $f_{rope}$ band. Then the maximum in this band is calculated and $x_{FFT-rope}$ indicators are obtained for the two pressure sensors. If both values exceed their respective thresholds the control signal is activated. For the present case, and based on the IEC 41 (Figure 15) the threshold value for PDT and PSC are considered respectively 0.1 and 0.15.

Figure 19b shows the operation of the control device (PLC). If the control signal is OFF, which means that the unit is far from a full load instability condition, the WGO reference signal pass through the device with no modification and goes directly to the governor. Nevertheless, when the control signal is active (onset of instability) the following actions have to be performed by the device. Let us consider a situation where the reference WGO signal has been set to a very high level (in order to increase the generating power) and for a determinate WGO the instability appears, which is rapidly detected by the protecting device. Just after it is detected, the two switches commute and the main block in Figure 19b is activated.

The instantaneous actual reference signal is recorded and reduced in a certain amount in order to bring the machine out from the unstable condition. This new version of the WGO signal (WGO_controlled_) is send to the governor, which mechanically acts on the wicket gates and at the same time it is send to the central system in order to modify the order of going to a higher power. The block waits a certain amount of time *(T_update_* is about few seconds) to perform a new correction (*Corr%*). These two parameters can be precisely determined with a test measuring the exiting of the instability (closing WGO). Note that too large *Corr (*%*)* value or too short *T_update_* will perform an excessive and maybe unnecessary correction of the WGO signal. 

If the control signal is turning back to an OFF-state before *T_update_*, then no further corrections on the signals are performed and the machine will keep working in the actual WGO after the switches are commuted again. If the control signal keeps on ON-state after *T_update_*, new reductions of the WGO signal will be performed until the machine exits the instability. 

As mentioned before, this device could be implemented in a generic unit by adjusting the different configuration parameters. To calibrate the device in another unit a similar test exploring the onset of instability may be necessary. For the present case, according to the analyzed test, the configuration parameters proposed (Figure 19) are shown in Table 3.

## 6. Conclusions

In this paper, the full load instability that occurs in some Francis turbines has been analyzed in a real 450 MW prototype by an extensive use and analysis of several types of sensors. In the actual electricity market, Francis turbines need to work in a wide operating range but phenomena such as the full load instability limit their effective operation. In this context, advanced sensor-based systems and an adequate signal processing can contribute to extend the operating range of the unit improving the use of renewable energies. 

Within the framework of a large EU project, the full load instability that occur in a real Francis turbine located in Canada has been experimentally analyzed. Several sensors (80 signals and 10 types of sensors) were installed and used to determine the dynamic behavior of the whole unit during the full load instability. After a first selection of the most suitable sensors discussed in [26], in this paper we perform a systematically approach to obtain an optimized acquisition strategy to detect the onset of this instability with good sensitivity and fast reaction time. 

It has been observed that some sensor indicators, obtained after an adequate signal processing, follow a linear correlation with the oscillating power. From all of them, the indicators obtained by the FFT analysis of the pressure sensors, located on the spiral case and draft tube, have been selected due to their good correlation characteristics, low acquisition frequency necessary and proved repeatability.

Finally, an economic and easy implementable protecting system based on the resulting optimized acquisition strategy is proposed. The proposed system acts when the onset of the instability is detected and brings the unit out of this unstable behavior by correcting the wicket gate opening. This system, which can be used in a generic Francis turbine with a similar instability, permits to extend the operating range of the unit by working close to the full load instability with a reasonable safety. 

## Figures and Tables

**Table 1:** Main characteristics of the sensors installed: ADT 10, AT 9, AGA 12, PSC 10, PDT 10, B07_01.

Sensor Name	Physical Unit	Location, Direction	Sensitivity
ADT 10	$\frac{m}{s^{2}}$	Draft tube wall Radial direction	$10\;\mathrm{mV}/\frac{m}{s^{2}}$
AT 9	$\frac{m}{s^{2}}$	Turbine guide bearing Radial direction	$10\;\mathrm{mV}/\frac{m}{s^{2}}$
AGA 12	$\frac{m}{s^{2}}$	Generator bearing Axial direction	$10\;\mathrm{mV}/\frac{m}{s^{2}}$
PSC 10	Pa	Draft tube pick-up pressure hole radial	400 mV/bar
PDT 10	Pa	Spiral casing pick-up pressure hole radial	400 mV/bar
B07_01	μm/m	Runner blade trailing edge	3265 mV/μm/m

**Table 2:** Summary of the correlation between relative indicators values and relative RMS of the oscillating power values.

Sensor	RMS			FFT			Envelope		
	Slope	Intercept	*R*^2^	Slope	Intercept	*R*^2^	Slope	Intercept	*R*^2^
ADT-low freq.	0.39	0.007	0.96	1.26	−0.26	0.65	0.61	0.13	0.83
ADT-high freq.	0.38	−0.008	0.94	1.26	−0.26	0.65	0.64	0.13	0.93
PDT	0.83	0.01	0.96	**0.63**	**0.1**	**0.94**	1.18	−0.13	0.85
PSC	0.80	0.003	0.96	**0.62**	**0.13**	**0.93**	1.14	0.01	0.82

**Table 3:** Configuration parameters for the protecting system.

Parameter	Present Case	Comments
Window length (FFT)	4 s	Less is not recommendable (resolution in frequency)
Refresh rate (FFT)	0.5 s	Enough small to have a reactive system
Sampling rate	10 samples/s	>5·$f_{rope}$
$f_{rope}\pm f_{tol}$	0.8125±0.3 Hz	To be determined experimentally
$x_{th-PSC},x_{th-PDT}$	0.15 and 0.1	To be determined experimentally according to IEC 41
*Corr* (%)	1%	To be determined experimentally testing the exit of the instability
$T_{update}$	2	To be determined experimentally testing the exit of the instability

## References

[1] Valero C., Egusquiza M., Egusquiza E., Presas A., Valentin D., Bossio M. (2017). Extension of Operating Range in Pump-Turbines. Influence of Head and Load. Energies.

[2] De Siervo F., De Leva F. (1976). Modern trends in selecting and designing Francis turbines. Water Power Dam Constr..

[3] Escaler X., Egusquiza E., Farhat M., Avellan F., Coussirat M. (2006). Detection of cavitation in hydraulic turbines. Mech. Syst. Signal Process..

[4] Kumar P., Saini R.P. (2010). Study of cavitation in hydro turbines—A review. Renew. Sustain. Energy Rev..

[5] Luo X.-W., Ji B., Tsujimoto Y. (2016). A review of cavitation in hydraulic machinery. J. Hydrodyn. Ser. B.

[6] Liu X., Luo Y., Wang Z. (2016). A review on fatigue damage mechanism in hydro turbines. Renew. Sustain. Energy Rev..

[7] Egusquiza E., Valero C., Huang X., Jou E., Guardo A., Rodriguez C. (2012). Failure investigation of a large pump-turbine runner. Eng. Fail. Anal..

[8] Gagnon M., Tahan A., Bocher P., Thibault D. (2013). On the fatigue reliability of hydroelectric Francis runners. Procedia Eng..

[9] Egusquiza E., Valero C., Estévez A., Guardo A., Coussirat M. (2011). Failures due to ingested bodies in hydraulic turbines. Eng. Fail. Anal..

[10] Egusquiza M., Egusquiza E., Valentin D., Valero C., Presas A. (2017). Failure investigation of a Pelton turbine runner. Eng. Fail. Anal..

[11] Rheingans W. (1940). Power swings in hydroelectric power plants. Trans. ASME.

[12] Valentín D., Presas A., Egusquiza E., Valero C., Egusquiza M., Bossio M. (2017). Power Swing Generated in Francis Turbines by Part Load and Overload Instabilities. Energies.

[13] Favrel A., Landry C., Müller A., Yamamoto K., Avellan F. (2014). Hydro-acoustic resonance behavior in presence of a precessing vortex rope: Observation of a lock-in phenomenon at part load Francis turbine operation. IOP Conf. Ser. Earth Environ. Sci..

[14] Favrel A., Müller A., Landry C., Gomes J., Yamamoto K., Avellan F. (2017). Dynamics of the precessing vortex rope and its interaction with the system at Francis turbines part load operating conditions. J. Phys. Conf. Ser..

[15] Favrel A., Müller A., Landry C., Yamamoto K., Avellan F. (2015). Study of the vortex-induced pressure excitation source in a Francis turbine draft tube by particle image velocimetry. Exp. Fluids.

[16] Favrel A., Müller A., Landry C., Yamamoto K., Avellan F. (2016). LDV survey of cavitation and resonance effect on the precessing vortex rope dynamics in the draft tube of Francis turbines. Exp. Fluids.

[17] Müller A., Favrel A., Landry C., Avellan F. (2017). Fluid–structure interaction mechanisms leading to dangerous power swings in Francis turbines at full load. J. Fluids Struct..

[18] Müller A., Favrel A., Landry C., Yamamoto K., Avellan F. (2014). On the physical mechanisms governing self-excited pressure surge in Francis turbines. IOP Conf. Ser. Earth Environ. Sci..

[19] Valero C., Egusquiza E., Presas A., Valentin D., Egusquiza M., Bossio M. (2017). Condition monitoring of a prototype turbine. Description of the system and main results. J. Phys. Conf. Ser..

[20] Egusquiza E., Valero C., Valentin D., Presas A., Rodriguez C.G. (2015). Condition monitoring of pump-turbines. New challenges. Measurement.

[21] Egusquiza M., Egusquiza E., Valero C., Presas A., Valentín D., Bossio M. (2018). Advanced condition monitoring of Pelton turbines. Measurement.

[22] HYdropower Plants PERformance and flexiBle Operation towards Lean Integration of New Renewable Energies. https://hyperbole.epfl.ch.

[23] Valentín D., Presas A., Bossio M., Egusquiza M., Egusquiza E., Valero C. (2018). Feasibility of Detecting Natural Frequencies of Hydraulic Turbines While in Operation, Using Strain Gauges. Sensors.

[24] Müller A., Dreyer M., Andreini N., Avellan F. (2013). Draft tube discharge fluctuation during self-sustained pressure surge: Fluorescent particle image velocimetry in two-phase flow. Exp. Fluids.

[25] Presas A., Valentin D., Egusquiza E., Valero C. (2017). Detection and analysis of part load and full load instabilities in a real Francis turbine prototype. J. Phys. Conf. Ser..

[26] Batllo A.P., Valentin D., Egusquiza M., Bossio M., Egusquiza E., Valero C. (2017). Optimized Use of Sensors to Detect Critical Full Load Instability in Large Hydraulic Turbines. Proceedings.

[27] Koutnik J., Krüger K., Pochyly F., Rudolf P., Haban V. On cavitating vortex rope form stability during Francis turbine part load operation. Proceedings of the IAHR International Meeting of the Workgroup on Cavitation and Dynamic Problems in Hydraulic Machinery and Systems.

[28] Ruprecht A., Helmrich T., Aschenbrenner T., Scherer T. Simulation of vortex rope in a turbine draft tube. Proceedings of the 22nd IAHR Symposium on Hydraulic Machinery and Systems.

[29] Oppenheim A.V. (1999). Discrete-Time Signal Processing.

[30] Presas A., Valentin D., Egusquiza E., Valero C., Seidel U. (2015). On the detection of natural frequencies and mode shapes of submerged rotating disk-like structures from the casing. Mech. Syst. Signal Process..

[31] Newland D.E. (1994). Wavelet Analysis of Vibration: Part 1—Theory. J. Vib. Acoust..

[32] Chui C.K. (1995). Wavelet Analysis and Its Applications.

[33] Wang Y., He Z., Zi Y. (2010). Enhancement of signal denoising and multiple fault signatures detecting in rotating machinery using dual-tree complex wavelet transform. Mech. Syst. Signal Process..

[34] Newland D.E. (2012). An Introduction to Random Vibrations, Spectral & Wavelet Analysis.

[35] Ewins D.J. (1984). Modal Testing Theory and Practice.

